# Fabrication and Properties of Carbon-Encapsulated Cobalt Nanoparticles over NaCl by CVD

**DOI:** 10.1186/s11671-016-1645-9

**Published:** 2016-09-27

**Authors:** Haipeng Li, Yue Li, Yongguang Zhang, Chunyong Liang, Hongshui Wang, Baoe Li, Desmond Adair, Zhumabay Bakenov

**Affiliations:** 1 School of Material Science and Engineering, Hebei University of Technology, Tianjin, 300130 China; 2 Research Institute for Energy Equipment Materials, Hebei University of Technology, Tianjin, 300130 China; 3 Tianjin Key Laboratory of Materials Laminating Fabrication and Interface Control Technology, Hebei University of Technology, Tianjin, 300130 China; 4 Institute of Batteries LLC, PI National Laboratory Astana System, Nazarbayev University, 53 Kabanbay Batyr Avenue, Astana, 010000 Kazakhstan

**Keywords:** Carbon-encapsulated metal nanoparticles, Sodium chloride, Chemical vapor deposition, Magnetic property

## Abstract

Carbon-encapsulated cobalt (Co@C) nanoparticles, with a tunable structure, were synthesized by chemical vapor deposition using Co nanoparticles as the catalyst and supported on a water-soluble substrate (sodium chloride), which was easily removed by washing and centrifugation. The influences of growth temperature and time on the structure and magnetic properties of the Co@C nanoparticles were systematically investigated. For different growth temperatures, the magnetic Co nanoparticles were encapsulated by different types of carbon layers, including amorphous carbon layers, graphitic layers, and carbon nanofibers. This inferred a close relationship between the structure of the carbon-encapsulated metal nanoparticles and the growth temperature. At a fixed growth temperature of 400 °C, prolonged growth time caused an increase in thickness of the carbon layers. The magnetic characterization indicated that the magnetic properties of the obtained Co@C nanoparticles depend not only on the graphitization but also on the thickness of the encapsulated carbon layer, which were easily controlled by the growth temperatures and times. Optimization of the synthesis process allowed achieving relatively high coercivity of the synthesized Co@C nanoparticles and enhancement of its ferromagnetic properties, which make this system promising as a magnetic material, particularly for high-density magnetic recording applications.

## Background

Magnetic metal nanoparticles (such as Co, Ni, and Fe) have attracted significant attention due to their enhanced electronic and magnetic properties. These properties have potential applications in ferrofluids, magnetic resonance imaging, and magnetic recording [1–4]. However, there are a series of problems that restrict their wide applications, such as oxidation, agglomeration, and instability in air. Carbon-encapsulated metal nanoparticles (CEMNPs), which may address the above problems, have attracted increasing interest due to their unique core/shell structure and properties [5, 6]. The role of carbon-encapsulating layers is to isolate the metal nanoparticles from each other, thus avoiding the problems caused by interactions among closely compacted magnetic units. Furthermore, stability and compatibility of the metal nanoparticles in application environments are enhanced by these protective carbon layers [7–9]. CEMNPs are thus endowed with new potential applications in the fields of catalytic synthesis, biomedicine, high-density magnetic data storage, and ferrofluids [10–12].

Various preparation methods, including arc discharge, chemical vapor deposition (CVD), pyrolysis, and explosions [13–17], have been developed to synthesize CEMNPs. Among these, CVD technique has received a particular attention due to its advantages such as a relatively low cost, potentially high yield, availability of raw materials, and a simple synthesis process [18]. However, there are still difficulties in achieving a controllable adjustment of magnetic properties of CEMNPs, particularly, due to a lack of knowledge on the relationship between the structure and magnetic property of CEMNPs. Additionally, ceramic materials such as Al_2_O_3_, SiC, and MgO are often used as the catalytic support to synthesize CEMNPs during the CVD process [19, 20]. However, the ceramic catalytic support creates additional problem because it is difficult to completely remove from the final products. This reduces the purity and affects the properties of CEMNPs. To solve this problem, utilization of a water-soluble material as a catalytic support instead of a ceramic one could be considered as a promising alternative. For example, Steigerwalt et al. [21] prepared graphitic carbon nanofibers with a catalyst supported by three different water-soluble compounds Na_4_SiO_4_, Na_2_SiO_3_, and Na_2_CO_3_. Zhao et al. [22] synthesized hierarchical porous carbon with a graphitic structure by a water-soluble template method. However, during the synthesis of CEMNPs by CVD method, a water-soluble support is seldom used because they usually have lower melting point and more unstable chemically compared with the ceramic supports. Therefore, it is still challenging to synthesize CEMNPs with high purity, a controllable structure, and good magnetic properties.

**Fig. 1:**
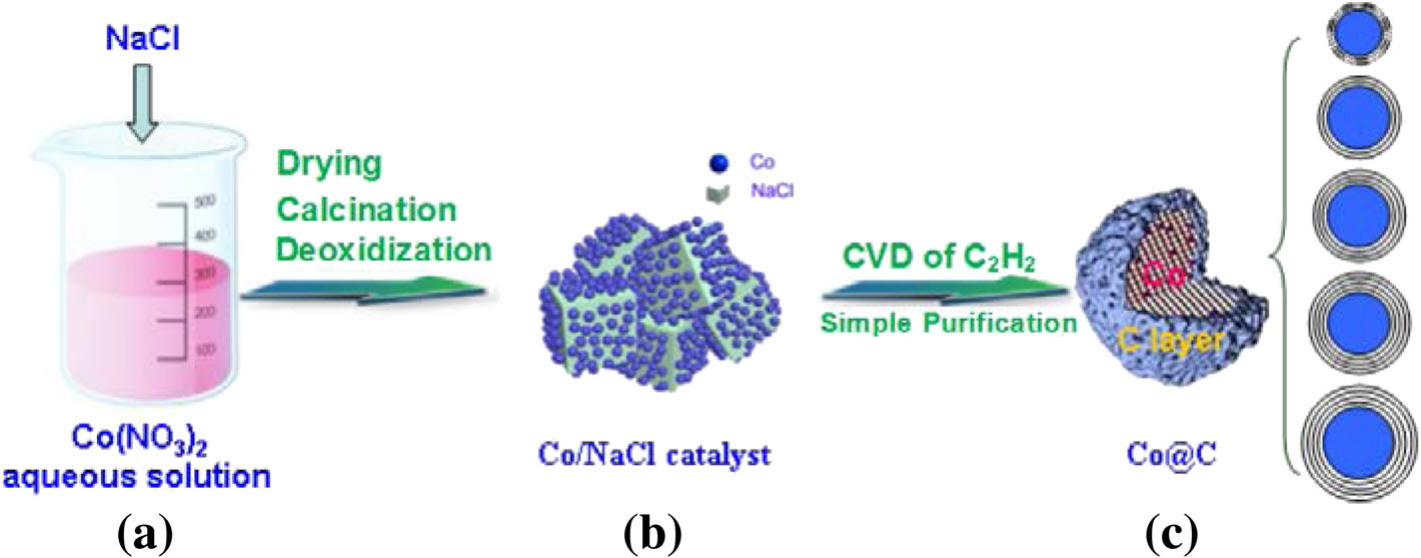
Scheme of fabrication processes of Co@C nanoparticles. **a** Preparation of catalyst precursor. **b** Formation of Co/NaCl catalyst. **c** Synthesis of Co@C nanoparticles with different structures

In this paper, we proposed a simple and economic approach to synthesize high-purity carbon-encapsulated cobalt (Co@C) nanoparticles using the CVD method with a water-soluble material as a catalytic support. As shown in Fig. 1, sodium chloride (NaCl) is used as a catalytic support, while cobalt and acetylene are employed as a graphitization catalyst and carbon precursor, respectively. Using this water-soluble material as a catalytic support instead of ceramic materials means that the catalytic support is removed by simple washing with water and centrifugation, which simplifies obtaining high-purity CEMNPs. Furthermore, the effect of synthesis temperature and time on the structure and magnetic properties of Co@C composites are discussed. By optimizing the synthesis process, the Co@C nanoparticles with a controllable structure, a graphitization degree, and magnetic properties were obtained. In particular, a high coercivity of the material enables its withstanding a larger degree of demagnetization, which is very useful in fabrication and application of Co@C nanocomposite in the area of high-density magnetic recording materials.

## Methods

### Preparation of Co@C Nanoparticles

NaCl powder (99.5 % purity) was added into 0.1 mol L^−1^ Co(NO_3_)_2_·6H_2_O (99.0 % purity) aqueous solution with constant stirring for 0.5 h, ensuring achieving a weight ratio of Co/NaCl remained at 1/98. The aqueous solution was dried at 60 °C for 72 h to acquire Co(NO_3_)_2_/NaCl mixture, which was then calcined in argon (99.99 % purity, 100 mL min^−1^) at 350 °C for 1 h, and the CoO/NaCl catalyst precursor was obtained. Synthesis of the Co@C nanoparticles included reducing the CoO/NaCl catalyst precursor in hydrogen gas (99.99 % purity, 100 mL min^−1^) at 350 °C for 2 h to acquire the Co/NaCl catalyst. The next step of the preparation process was a heat treatment of the sample in mixture gas of acetylene (99.9 % purity, 30 mL min^−1^)/argon (420 mL min^−1^) at different growth temperatures (350, 400, 450, and 500 °C) for 60 min. In addition, a similar synthesis process was performed at 400 °C for different growth times (5, 15, and 30 min). Black powders were obtained after the reactor was cooled to room temperature in an argon atmosphere (100 mL min^−1^).

### Purification of Co@C Nanoparticles

To purify the Co@C nanoparticles, the prepared black powders were thoroughly washed by ultrasonication in distilled water in a round-bottomed flask for 1 h. The precipitate was separated from liquid phase using a high-speed centrifuge (Evolution RC; Thermo Electron LED GmbH) at 8000 rpm for 30 min. The resulting solid precipitates were dried in a dryer at 80 °C for 24 h, and the final Co@C nanoparticles were obtained.

### Characterization

Surface morphology and microstructure of the Co@C nanoparticles were characterized using field emission scanning electron microscope (FE-SEM; Hitachi S-4800) and high-resolution transmission electron microscope (HRTEM; Philips Tecnai G2 F20). X-ray diffraction (XRD) patterns of the Co@C nanoparticles were recorded on a powder X-ray diffractometer (Rigaku D/max 2000V/pc) using CuKα radiation within the angle range of 20°–90° (2θ) with a 0.02° step. The interplanar spacing of the graphite layer was calculated using the Digital Micrograph software. Raman spectroscopy was performed using the 532-nm line of Ar^+^ laser as the excitation source on a Thermo Fisher DXR Raman Microscope. Thermal properties of the samples were investigated using thermogravimetric analysis (TGA, TA Instruments SDT Q600 TGA). The samples in TGA were analyzed in platinum pans at a heating rate of 10 °C min^−1^ from 50 to 700 °C in air with a flow rate of 150 mL min^−1^. The magnetic properties of the samples were measured using a vibrating sample magnetometer (VSM; Lakeshore 7407) at room temperature.

## Results and Discussion

**Fig. 2:**
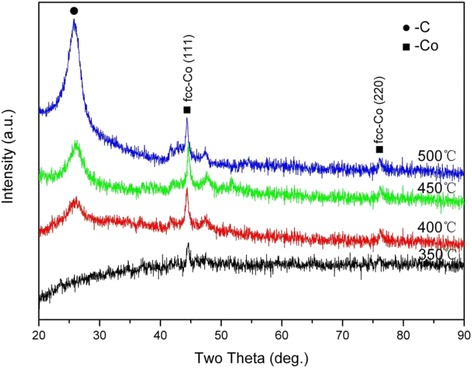
XRD patterns of purified Co@C nanoparticles grown at different temperatures

Figure 2 shows the XRD patterns of the Co@C nanoparticles grown at different CVD temperatures. There were no characteristic peaks observed belonging to the NaCl crystal, inferring the complete removal of NaCl from the Co@C nanoparticles. When the growth temperature was 350 °C, only the peaks at 44.2° and 75.8°, identified as the (111) and (220) planes of β-Co with a face-centered cubic (fcc) structure, were observable. No obvious diffraction peak of graphitic carbon appeared due to the low content and crystallinity of carbon deposited in this sample. When the growth temperatures were at 400, 450, and 500 °C, a diffraction peak appeared around 26.2°, which can be assigned to the (002) planes of a hexagonal graphite structure. The broadened diffraction peak suggests that the resultant carbon products should have more structural defects than ideal graphitic carbon. With a temperature rise, this peak becomes more symmetrical and narrow, indicating the higher crystallinity of the carbon product obtained at the higher temperatures. Furthermore, no compound phase of carbon and cobalt was found in the samples.

**Fig. 3:**
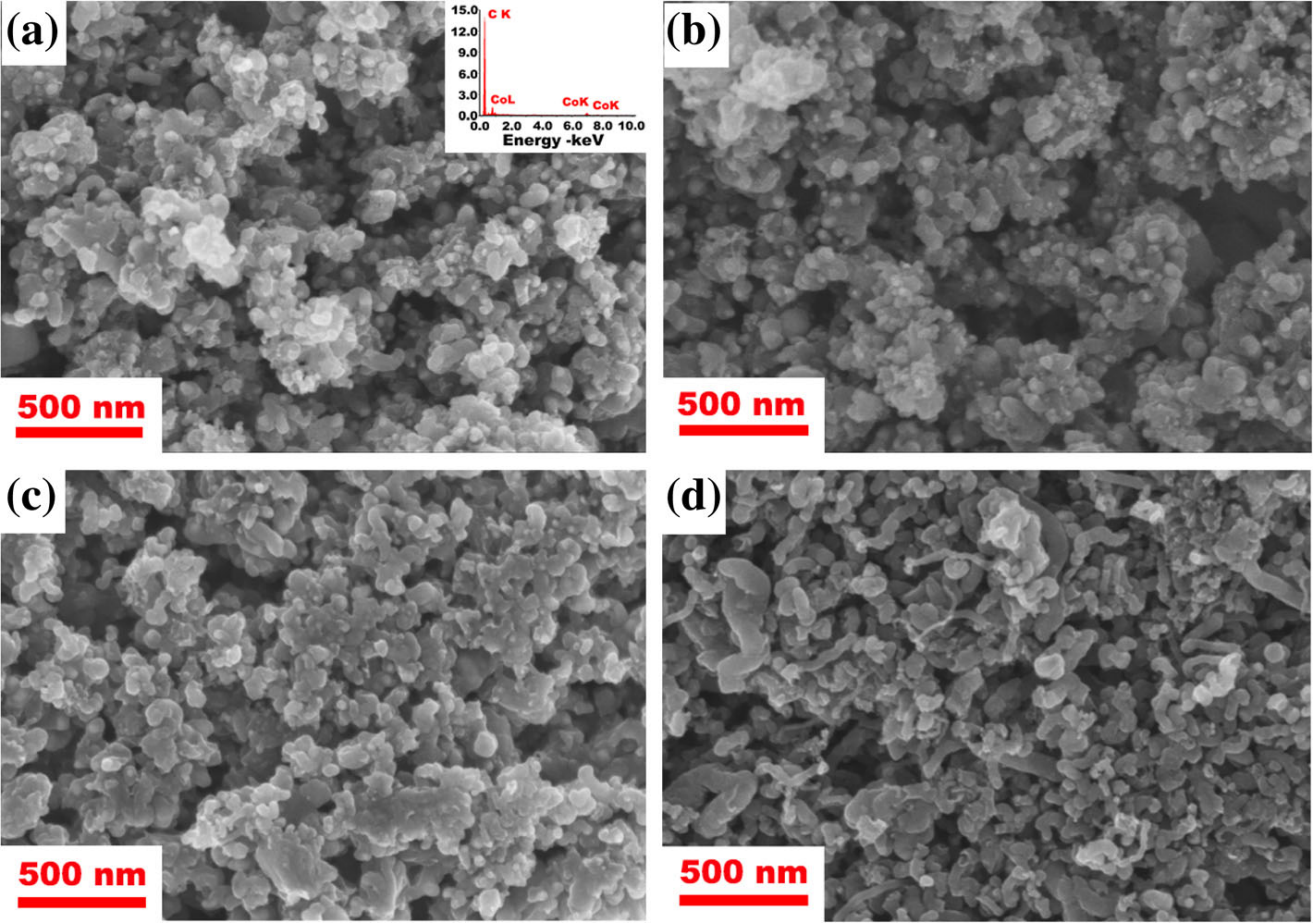
SEM images of purified Co@C nanoparticles grown at **a** 350, **b** 400, **c** 450, and **d** 500 °C for 60 min

The morphology and structure of the Co@C nanoparticles were characterized by SEM and TEM. Figure 3 shows the typical SEM images of the purified Co@C nanoparticles synthesized at different temperatures. It was found that the particle size and shape of the Co@C nanoparticles vary with the preparation temperature. For the product grown at 350 °C, as shown in Fig. 3a, the synthesized Co@C nanoparticles are spherical and have a diameter in the range of 30–40 nm. The surfaces of the Co@C nanoparticles are clean and smooth, and no other type of carbon nanostructures could be observed, implying high purity of the Co@C nanoparticles. In addition, the EDX spectrum (inset in Fig. 3a) revealed that the dominating ingredients of the purified samples were C and Co, without the presence of Na and Cl. The spectrum also indicated that the NaCl used as the catalytic support was easily removed by simple washing and centrifugation. When the growth temperature was increased to 400 and 450 °C, as shown in Fig. 3b, c, a large number of the Co@C nanoparticles with a uniform size were synthesized. Their diameters were larger than that of the Co@C nanoparticles grown at 350 °C. This is because the higher growth temperatures would increase the chance of collision fusion among Co metal particles, including the reactivity of Co catalyst and C atoms, which would result in the increase of particle size [23, 24]. Therefore, the rise of growth temperature promoted the formation and growth of Co@C nanoparticles. However, when further increasing growth temperature to 500 °C, many carbon nanofibers (CNFs) were observed (See Fig. 3d), which reduces the purity of the Co@C nanoparticles.

**Fig. 4:**
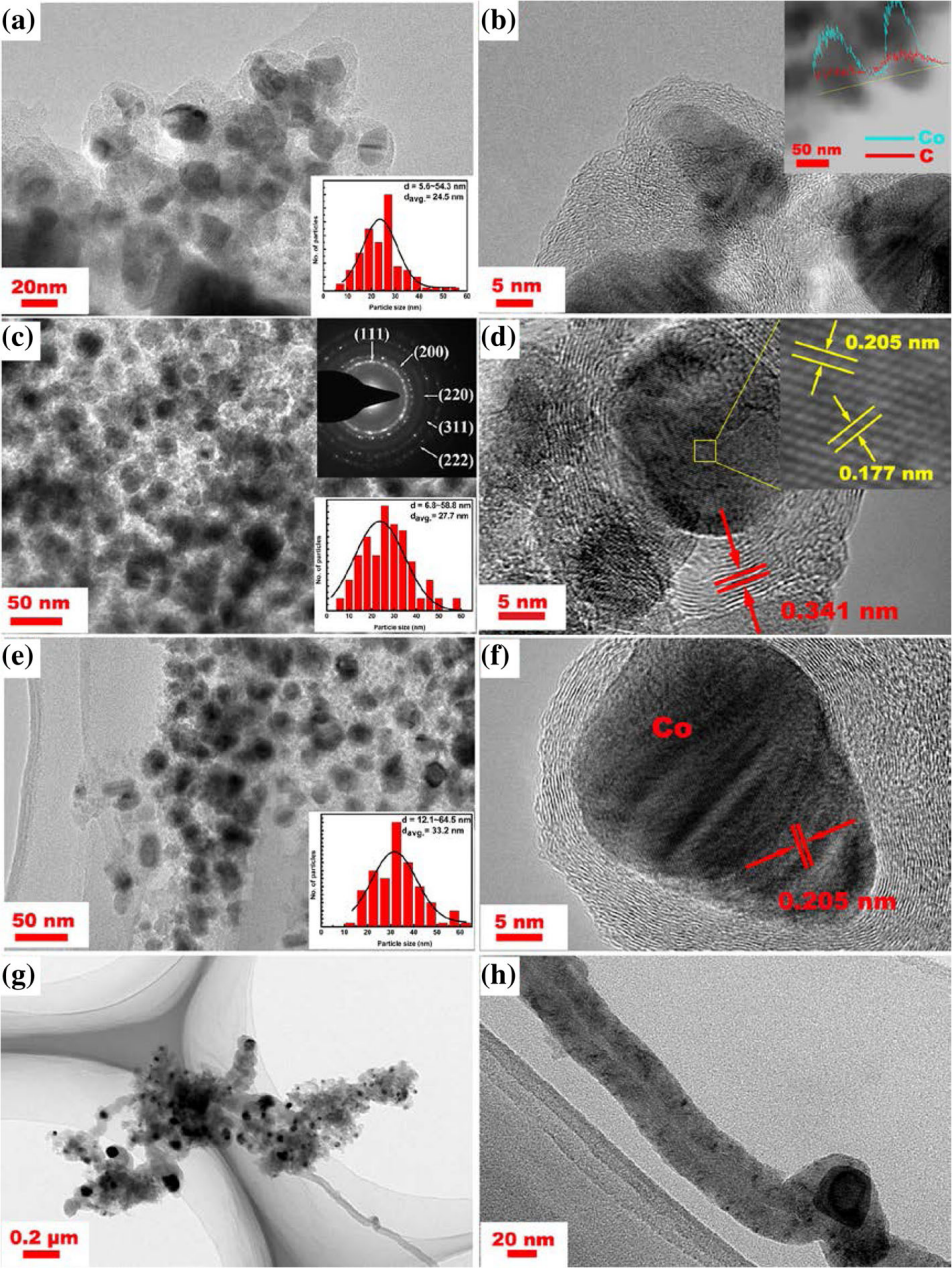
TEM images of purified Co@C nanoparticles grown at **a**, **b** 350; **c**, **d** 400; **e**, **f** 450; and **g**, **h** 500 °C for 60 min. (*Insets* of **a**, **c**, **e**: the diameter distribution of Co nanoparticles based on the TEM analysis; *inset* of **b**: the EDS line scanning analysis of Co@C nanoparticles; *inset* of **c**: the diffraction pattern)

The TEM images of the Co@C nanoparticles grown at different temperatures are presented in Fig. 4. Figure 4a, c, e shows the low-magnification TEM images of the Co@C nanoparticles, which indicate that the samples are composed of single nanoparticles, without any carbon nanotubes (CNTs) or CNFs. The nanoparticles consist of outer graphitic layers and inner Co nanoparticles, which are consistent with those prepared by the DC arc discharge method [25]. At a lower growth temperature of 350 °C, as shown in Fig. 4a, the synthesized product exhibit the core/shell structure of CEMNPs. According to the particle size distribution (inset of Fig. 4a) obtained from the TEM analysis, the average diameter of the Co nanoparticles in the Co@C composite structure is about 24.5 nm with the size distribution range of 5.6~54.3 nm. A typical Co@C nanoparticle synthesized at 350 °C is shown in Fig. 4b. Similar to amorphous carbon, the carbon-encapsulated layers keep the state of short-range order and long-range disorder and have many structural defects. This is due to the low catalytic activity of the Co nanoparticles and reactivity of carbon atoms at a lower growth temperature. From the EDS line scanning analysis (inset of Fig. 4b) under TEM, it was further determined that the core/shell nanoparticles are composed of Co and C. At higher growth temperatures of 400 and 450 °C, the size of the Co nanoparticles slightly increased and the degree of graphitization of carbon-encapsulated layers was improved. As shown in Fig. 4c, e, many Co@C nanoparticles with a uniform size were obtained. For 400 and 450 °C, the size distribution (insets of Fig. 4c, e) shows that the core size is distributed in the range of 6.8~58.8 and 12.1~64.5 nm, with the average diameter of 27.7 and 33.2 nm, respectively. Compared with that of 350 °C, the increasing of particle size was due to the agglomeration and combination of the Co nanoparticles at higher temperature. The diffraction patterns (inset of Fig. 4c) also confirm the good crystal structure of Co nanoparticles and can be ascribed to the (111), (200), (220), (311), and (222) planes of fcc Co. As shown in Fig. 4d, f, the carbon shells outside the Co nanoparticles have a crystalline graphite structure and consist of multi-shell graphite carbon. The carbon-encapsulated layers are clear and integrated, with a thickness of 5–10 nm, which can provide effective protection from corrosion to the Co nanoparticles and keep their physical and chemical stability. The interplanar spacing between the graphitic layers is approximately 0.341 nm, similar to the ideal (002) interplanar spacing (0.34 nm) of graphite carbon. The encapsulated Co nanoparticle has a clear lattice fringe, which can be indexed to the (111) and (200) planes of fcc cobalt with the d-spacing of 0.205 and 0.177 nm, respectively. However, when the growth temperature was increased to 500 °C, the Co nanoparticles with higher catalytic activity cause the formation of CNFs, as shown in Fig. 4g, h. Thus, CVD temperature is critical to control the size, structure, and purity of Co@C nanoparticles grown on the NaCl support.

**Fig. 5:**
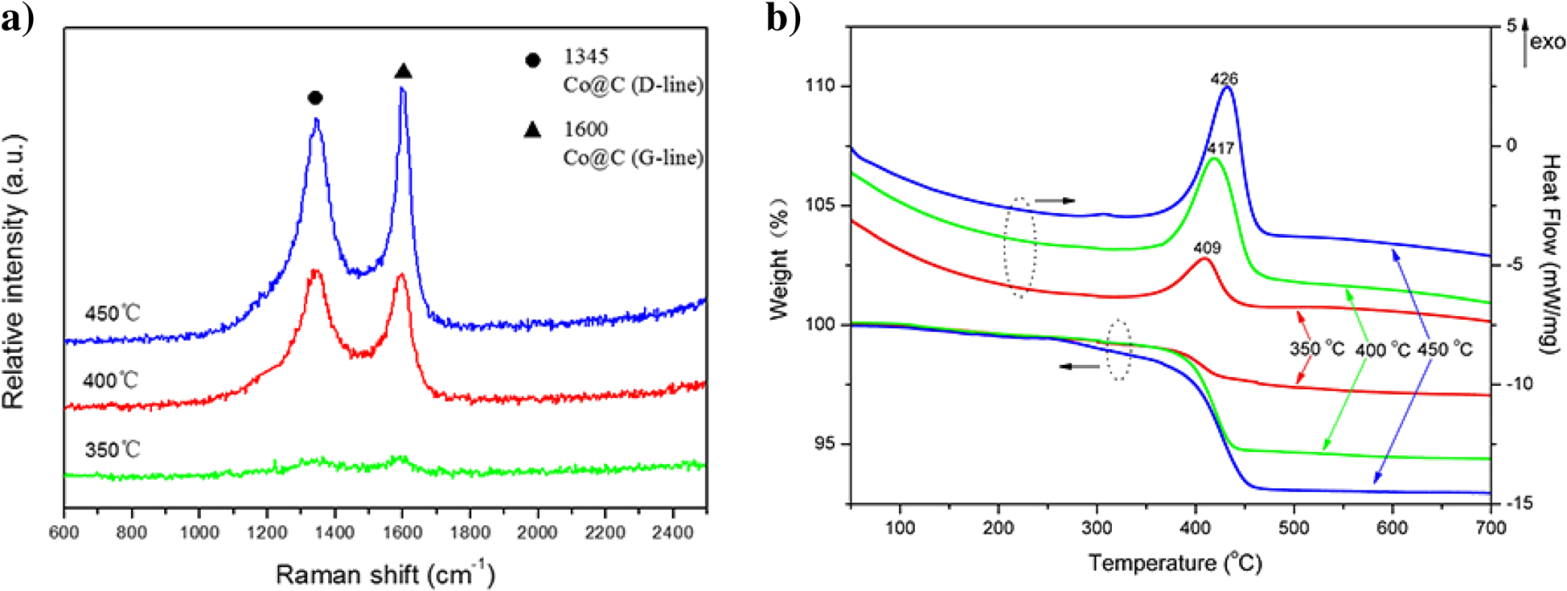
**a** Raman spectra and **b** TGA-DSC curves of Co@C nanoparticles

Figure 5a shows the Raman spectrum of Co@C nanoparticles grown at 350, 400, and 450 °C. No remarkable Raman band is observed for the sample grown at 350 °C, while both other samples grown at 400 and 450 °C exhibit two Raman bands at ~1345 cm^−1^ (D band) and ~1600 cm^−1^ (G band), which are associated with the vibrations of carbon atoms with dangling bonds for the in-plane terminations of disordered graphite (D) and the vibrations in all sp^2^-bonded carbon atoms in a 2D hexagonal lattice (G), respectively. The relative intensity values (*I*_D_/*I*_G_) for the Co@C nanoparticles grown at 400 and 450 °C are 1.02 and 0.90, respectively. The rise of growth temperature has resulted in an obvious improvement in the degree of graphitization. Figure 5b shows the TGA-DSC curves of the Co@C nanoparticles grown at different temperatures. During the heating process, all samples exhibit a similar oxidation behavior and a single-step degradation. The samples show a slight mass loss at the initial heating stage, possibly due to the presence of water in the samples [26]. Near 400 °C, a sharp weight loss, associated with exothermic process, was observed, which might correspond to the oxidation of carbon-encapsulated layers. At the growth temperature of 350 °C, the weight loss appears in the temperature interval of 370–430 °C. Due to its low content of carbon, the weight loss of the sample is smaller than the other two samples. Approximately 97 % of the sample remained behind after performing TGA up to 700 °C. In the case of the Co@C nanoparticles grown at 400 °C, the weight loss increases sharply and the sample stops losing weight near 450 °C, with approximately 94.5 % of the sample still remaining. When the sample growth temperature is 450 °C, the Co@C nanoparticles show a slightly higher rapid oxidation temperature and stop losing weight near 470 °C, showing that they possess better thermostability. Finally, approximately 93 % of the sample remained. From the above experimental results, it is known that the Co@C nanoparticles grown at 450 °C possess a much higher degree of crystalline perfection and oxidation stability.

**Fig. 6:**
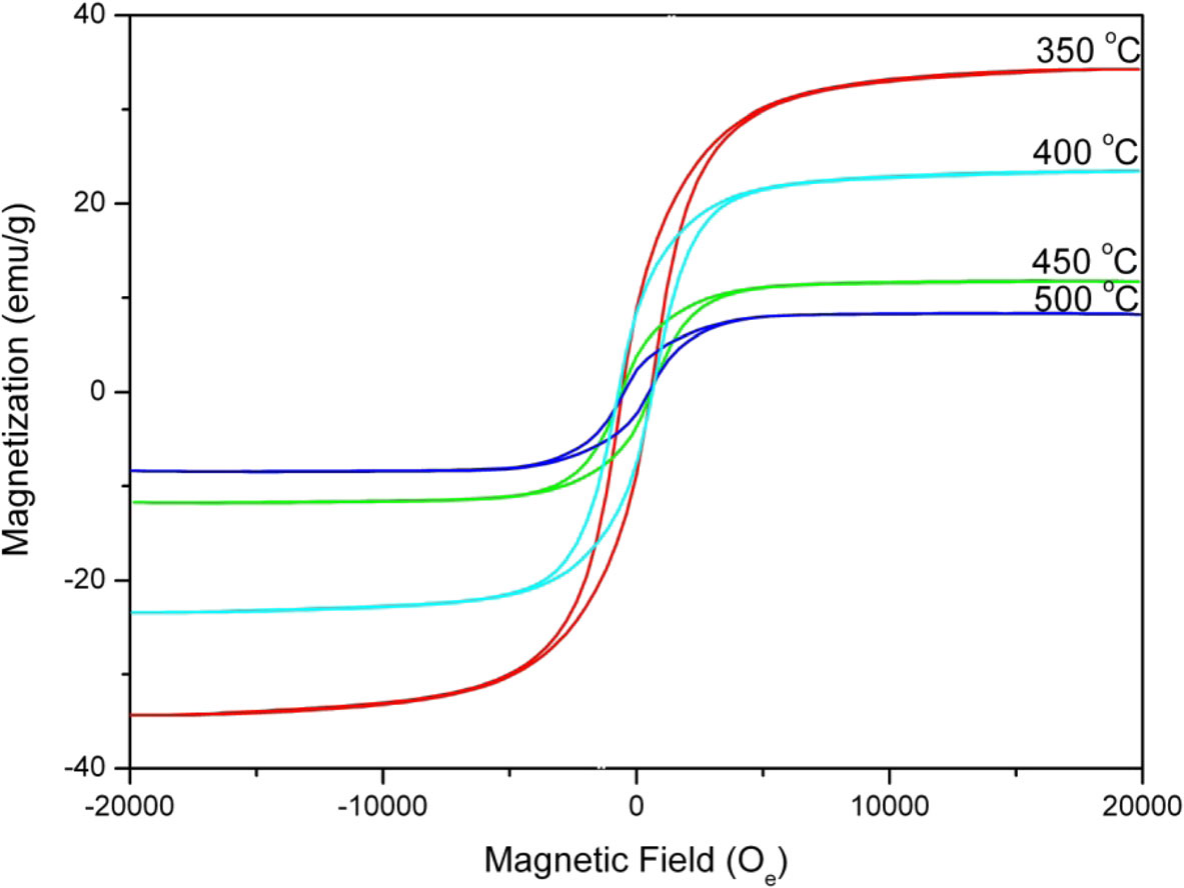
Hysteresis loops of Co@C nanoparticles grown at different temperatures for 60 min

Magnetic properties of the purified Co@C nanoparticles measured at room temperature are shown in Fig. 6 and Table 1. For the magnetic nanoparticles, the magnetic properties, especially the saturation magnetization (*M*_s_) and coercive force (*H*_c_), are dependent upon chemical composition and particle size [27]. The *M*_s_ and *H*_c_ of the Co@C nanoparticles grown at 350~450 °C are in the ranges 11.774~34.271 emu g^−1^ and 568.25~636.73 O_e_, respectively. These are higher than the reported values for the Co@C nanoparticles with similar composition and size [28, 29]. The ratios of the remanence to saturation magnetization (*M*_r_/*M*_s_) are in the range 0.253~0.357, indicating enhanced ferromagnetism of the Co@C nanoparticles at room temperature. The results of magnetic measurement also show that the magnetic properties of the Co@C nanoparticles vary with the temperatures of the CVD process. Through observation, the carbon encapsulation process keeps the squareness of the hysteresis loops. However, with an increase in the growth temperature, the squareness is progressively compressed, indicating a further reduction of the ferromagnetic nature of Co@C nanoparticles. Furthermore, it is evident that the values of *M*_s_ of all the Co@C nanoparticles are lower than that of pure Co (*M*_s_ = 120.79 emu g^−1^). The decrease of *M*_s_ may be attributed to the fact that the Co nanoparticles are entirely encapsulated by the graphitized carbon layers, which belong to the diamagnetic substance for *M*_s_ and weaken the effect [27, 30]. As the temperature increases, the inclusive phases of carbon in the Co@C nanoparticles rise. The diamagnetic contribution of carbon results in the reduction of the ferromagnetic nature of the Co@C nanoparticles. In particular, it should be mentioned that with the rise of synthesis temperature, the increase of crystallinity and particle size of Co as cores should cause the improvement of *M*_s_. Therefore, the decrease of *M*_s_ indicates that the magnetic properties of Co@C nanoparticles are affected synthetically by C and Co and mainly depend on the non-magnetic carbon shells in this study. In addition, for the Co nanoparticles, above the critical size that results in superparamagnetism, *H*_c_ will increase with a decrease in particle size. The *H*_c_ values of the grown Co@C nanoparticles are much larger than that of the pure Co (*H*_*c*_ = 122.86 O_e_), which is the result of size effect. For the Co@C nanoparticles grown at 400 °C, the high *H*_c_ value enables them to bear a larger degree of demagnetization, which is necessary for application in high-density magnetic recording materials.

**Table 1:** Magnetic properties of pure Co and Co@C nanoparticles grown at different temperatures for 60 min

Materials	*M*_s_ (emu g^−1^)	*M*_r_ (emu g^−1^)	*M*_r_/*M*_s_	*H*_c_ (O_e_)
Pure Co	120.79	4.1016	0.034	122.86
Co@C nanoparticles grown at 350 °C	34.271	8.681	0.253	568.25
Co@C nanoparticles grown at 400 °C	23.455	8.3796	0.357	636.73
Co@C nanoparticles grown at 450 °C	11.774	3.7637	0.320	589.94
Co@C nanoparticles and CNFs grown at 500 °C	8.338	2.2586	0.271	509.68

**Fig. 7:**
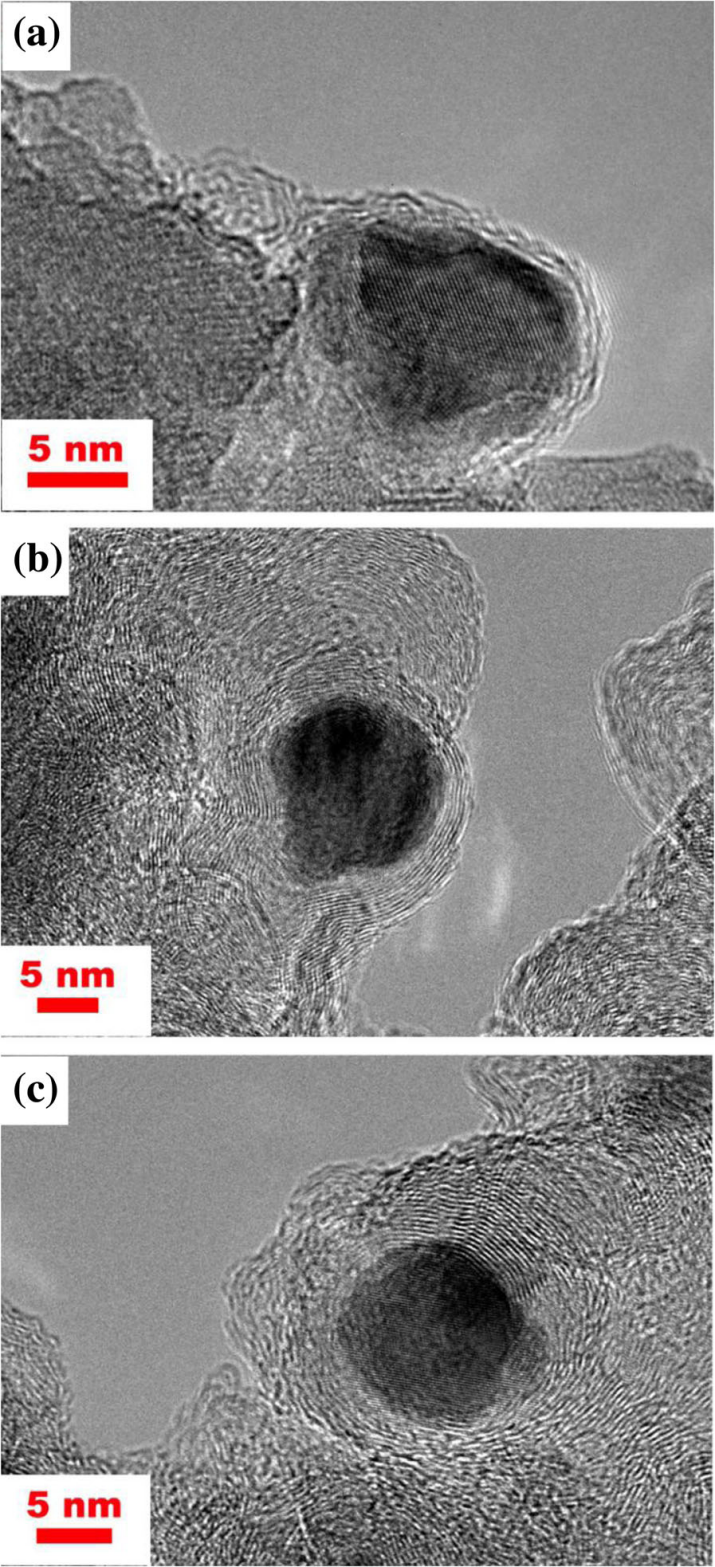
TEM images of the Co@C nanoparticles grown at 400 °C for **a** 5, **b** 15, and **c** 30 min

**Fig. 8:**
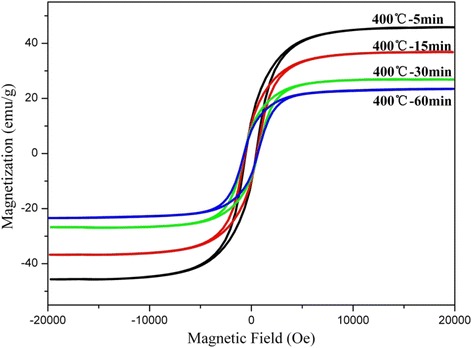
Hysteresis loops of Co@C nanoparticles grown at 400 °C for different times

In order to further reveal the relationship between the thickness of the graphite layer and magnetic properties, the Co@C nanoparticles were grown at 400 °C for different times. TEM images (Fig. 7) show the changes in the morphology of the Co@C nanoparticles when varying the growth time from 5 to 30 min at 400 °C. When prolonging the grown time, the size of Co nanoparticles changes a little, but the thickness of carbon layers increases. The amount of the graphitic layers increases from two layers for 5 min (as shown in Fig. 7a) to 17 layers for 30 min (as shown in Fig. 7c). From comparison of Fig. 7c with Fig. 4d, i.e., by prolonging the growth time further, the thickness change of carbon layers is not apparent. Figure 8 and Table 2 indicate the relationship between the magnetic properties and the growth time (layer thickness). It can be noted that the values of *M*_s_ decrease and the values of *H*_c_ increase by prolonging the growth time. Thus, the growth time (i.e., the thickness of carbon layers) can affect the magnetic properties of the Co@C nanoparticles greatly. The decrease of *M*_s_ can be explained by the rise of carbon in the samples as already mentioned. The change of *H*_c_ is largely due to the increasing of the carbon layer thickness, which can weaken the interaction of the dipoles in the Co nanoparticles and improve the coercivity [31, 32]. In addition, it can also be shown that the value of *H*_c_ is only improved by 0.89 % when prolonging the growth time from 30 to 60 min. Therefore, the attempt to further increase *H*_c_ by prolonging the growth time may be impracticable. In our study, the growth temperature of 400 °C and the growth time of 30 min are the feasible conditions to obtain Co@C nanoparticles with a higher coercive force.

**Table 2:** Magnetic properties of Co@C nanoparticles grown at 400 °C for different times

Materials	*M*_s_ (emu g^−1^)	*M*_r_ (emu g^−1^)	*M*_r_/*M*_s_	*H*_c_ (O_e_)
Co@C nanoparticles grown at 400 °C for 5 min	45.869	11.855	0.258	479.82
Co@C nanoparticles grown at 400 °C for 15 min	36.862	10.596	0.287	546.69
Co@C nanoparticles grown at 400 °C for 30 min	26.938	9.11371	0.338	631.11

## Conclusions

The Co@C nanoparticles were synthesized using NaCl as the catalytic support at different growth temperatures and times using the CVD method. By using NaCl as the catalytic support, the purification of the Co@C nanoparticles is easier and consists of only simple washing and centrifugation. At low growth temperatures (350 °C), the Co nanoparticles are encapsulated in amorphous carbon shells, which have a lower graphitization degree and oxidation stability. At 400 and 450 °C, the Co@C nanoparticles with well-ordered graphitic shells and good thermal oxidation stability are obtained. A higher growth temperature (500 °C) reduces the purity of the Co@C nanoparticles due to the formation of CNFs. The growth time is also a key factor in controlling the thickness of carbon layers. The magnetic characterization shows that the grown Co@C nanoparticles possess relatively large coercive force and good ferromagnetism, and the magnetic properties are closely related to their structures. Thus, by controlling the growth temperature and time of CVD in this process, the purity, morphology, structure, and magnetic property of Co@C nanoparticles could be easily tuned. Meanwhile, the high coercivity of the Co@C nanoparticles enabled the material to bear a larger degree of demagnetization, which indicates their great application potential in the field of high-density magnetic recording materials.

## Abbreviations

CEMNPs: Carbon-encapsulated metal nanoparticles
CNFs: Carbon nanofibers
CNTs: Carbon nanotubes
Co@C: Carbon-encapsulated cobalt
CVD: Chemical vapor deposition
fcc: Face-centered cubic
FE-SEM: Field emission scanning electron microscope
*H*_c_: Coercive force
HRTEM: High-resolution transmission electron microscope
*M*_s_: Saturation magnetization
NaCl: Sodium chloride
TGA: Thermogravimetric analysis
XRD: X-ray diffraction
